# Influence of including or excluding endocardial borders on left ventricular wall thickness measurements in cats

**DOI:** 10.1093/jvimsj/aalag224

**Published:** 2026-10-01

**Authors:** Maximiliane G Sehn, Yuen Yee Cheuk, Jose Novo Matos, David Brodbelt, Virginia Luis Fuentes

**Affiliations:** Clinic for Small Animals, University of Veterinary Medicine Hannover Foundation, 30559 Hannover, Germany; Department of Clinical Science and Services, The Royal Veterinary College, Hatfield AL9 7TA, United Kingdom; Department of Clinical Science and Services, The Royal Veterinary College, Hatfield AL9 7TA, United Kingdom; The Queens Veterinary School Hospital, University of Cambridge, Cambridge CB3 0ES, United Kingdom; Department of Clinical Science and Services, The Royal Veterinary College, Hatfield AL9 7TA, United Kingdom; Department of Clinical Science and Services, The Royal Veterinary College, Hatfield AL9 7TA, United Kingdom

**Keywords:** echocardiography, feline, HCM, endocardial border, variability

## Abstract

**Background:**

Consensus is lacking on which endocardial borders to include when measuring left ventricular wall thickness (LVWT) in cats.

**Hypothesis/Objectives:**

The number of endocardial borders included can influence whether an individual cat is classified as having hypertrophic cardiomyopathy (HCM) or not.

**Animals:**

Fifty-two cats that previously were diagnosed as either healthy, equivocal for HCM, or having an HCM phenotype.

**Methods:**

Single-center retrospective cross-sectional study. Three observers measured LVWT using 4 different LVWT measurement protocols: leading edge-to-leading edge (L-L); leading edge-to-trailing edge, trailing edge-to-leading edge, and trailing edge-to-trailing edge for the interventricular septum and L-L and trailing edge-to-leading edge methods for the left ventricular free wall (LVFW). Measurements were made using stored echocardiographic images on 2 occasions by 2 observers. A Friedman test and post-hoc Wilcoxon signed rank test with Bonferroni correction were performed to detect differences between measurement techniques.

**Results:**

Interventricular septum (IVS) wall thickness was significantly larger for leading-edge-to-trailing-edge measurement (median and [interquartile range (IQR)]: 5.5 [3.8-7.2] millimeters) compared with other methods (*P* < .001). The trailing-edge-to-leading-edge method produced significantly smaller results (4.3 [IQR, 3.4-6.2] millimeters for the interventricular septum and 4.8 [IQR, 3.4-6.2] millimeters for the LVFW) compared with other methods (*P* < .001). Median IVS thickness measured as leading-edge-to-trailing-edge was 1.2 millimeters larger than when measured as trailing-edge-to-leading-edge.

**Conclusions and clinical importance:**

LVWT varies depending on the number of endocardial borders included. The number of endocardial borders included in LVWT measurements should be standardized.

## Introduction

Hypertrophic cardiomyopathy (HCM) is the most common cardiac disease in cats with a reported prevalence of 15%^1^ to 19.6%.^2^ The clinical diagnosis of HCM in cats is principally based on echocardiographic measurement of increased left ventricular wall thickness (LVWT).^1^

Cats with LVWT < 5.0 millimeters (mm) generally are considered normal. Cats with LVWT ≥ 6 mm generally are considered to have the HCM phenotype.^1^^,^^3^ Cats with an LVWT between 5.0 to 5.9 mm are in an equivocal range, and other features, such as papillary muscle hypertrophy, end-systolic cavity obliteration, papillary muscle and mitral leaflet abnormalities, systolic anterior motion of the mitral valve, abnormal cardiac geometry, presence of spontaneous echo-contrast or thrombi, and regional wall motion abnormalities should be taken into account.^3^ Differences between different diagnoses are based on as little as 1 mm. Diagnosis of HCM phenotype can lead to further follow-up echocardiograms, breeding restrictions in pedigreed cats, and adverse financial and emotional effects on owners. Although the differences in scale of LVWT measurements can be small, the clinical impact is substantial in terms of management of affected cats and owner anxiety. Consequently, precise measurement of LVWT is essential for phenotyping cats,^4^ and the method should be standardized.

Echocardiographic assessment of LVWT in cats can be challenging, requiring acquisition of optimized end-diastolic right parasternal long and short axis views. Additional challenges include the measurement itself, because tricuspid valve chordae, moderator bands, papillary muscles, and false tendons should be excluded. Approaches to measurement of the interventricular septum have varied widely from including 2, 1 or no endocardial borders.^4^ M-mode echocardiography typically uses a leading edge-to-leading edge (L-L) measurement technique.^5–7^ There is little consensus on which endocardial borders to include when measuring two-dimensional (2D) images of the LVWT.^4^^,^^8^ The American College of Veterinary Internal Medicine (ACVIM) consensus statement guidelines on cardiomyopathies in cats recommend a leading edge-to-trailing edge (L-T) technique for interventricular septum (IVS) measurements, and a L-L technique for the left ventricular free wall (LVFW).^1^^,^^9^

Previous studies have reported the reproducibility of LVWT measurements in cats when applying one measurement technique.^8^^,^^10^^,^^11^ In our study, we aimed to report the effect of including endocardial borders on 2D LVWT measurements in both healthy and affected cats. We also aimed to analyze reliability of LVWT measurements, including intra-observer repeatability and inter-observer reproducibility. We hypothesized that the number of endocardial borders included would influence whether an individual cat would be classified as HCM-affected or non-affected.

## Materials and methods

### Animals

The database of a single center was retrospectively searched for echocardiograms of cats performed between January 1, 2023 and January 31, 2024.

Based on the diagnosis at presentation, 3 groups were created: healthy cats with LVWT under 5.5 mm, an equivocal group with LVWT between 5.5 and 5.9 mm, and an HCM group with LVWT of at least 6 mm.

### Echocardiographic measurements

Wall thickness of 52 cats was measured by 3 observers: a veterinary student who received a brief period of training on LVWT measurement, a second-year resident in veterinary cardiology, and a board-certified cardiologist. End-diastolic interventricular septum (IVS) thickness and LVFW thickness were measured on 2D images in a right parasternal short axis view at the mid-papillary muscle level.^12^ This view is standardized and would be appropriate for M-mode measurement. Measurements were exclusively performed at the end of diastole (Figure 1). Measurements of 3 cardiac cycles were performed for each measurement method. The methods for IVS thickness measurement consisted of measuring from L-L, L-T, trailing edge-to-leading edge (T-L), and trailing edge-to-trailing edge (T-T, Figure 2). For the LVFW, measurements were performed using the L-L and T-L method (Figure 3). The cardiology resident and veterinary student repeated measurements of the same patients and on the same views at least 14 days later, while being blinded to previous measurements.

**Figure 1:**
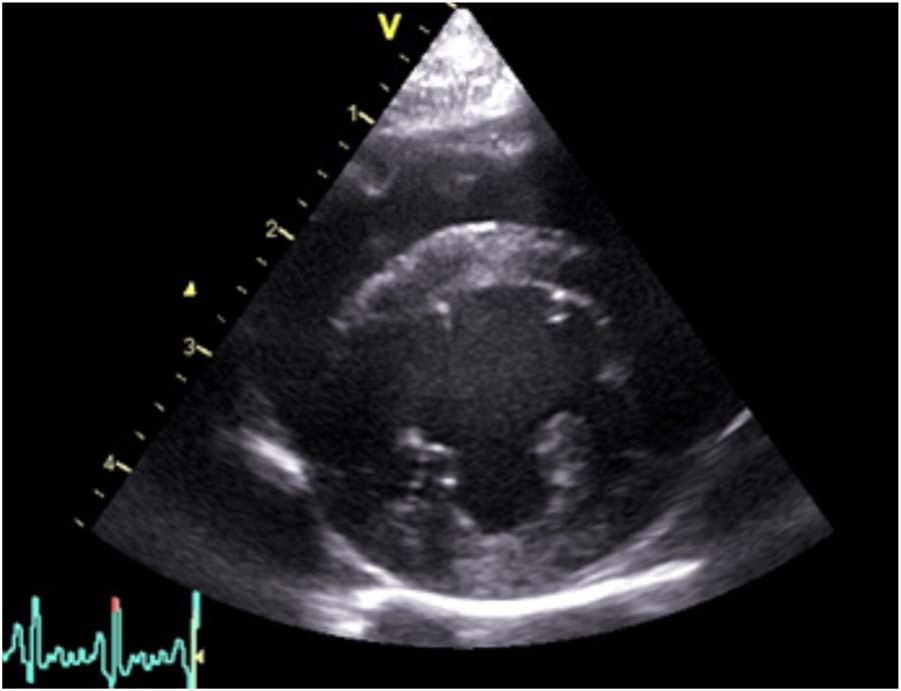
Echocardiographic right parasternal short-axis view on the level of the papillary muscles with concurrent ECG.

**Figure 2:**
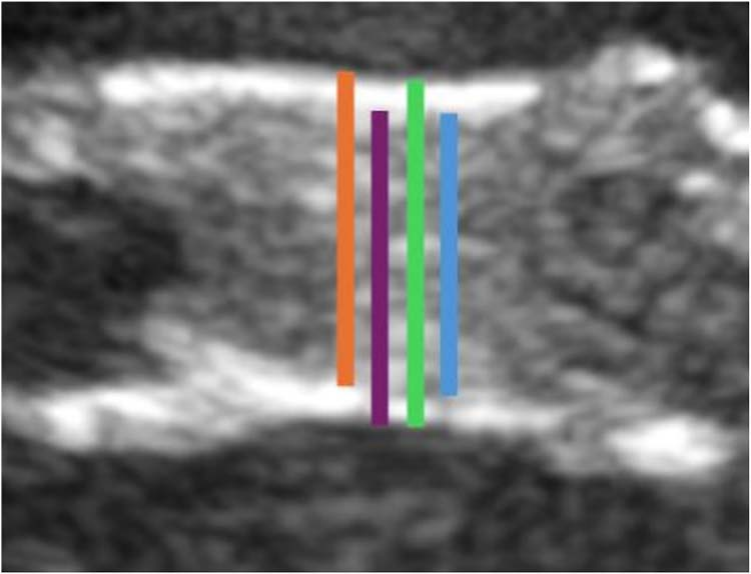
Magnification of the interventricular septum showing the different measurement techniques. Orange line (leftmost line): leading edge-to-leading edge; purple line (second line from the left): trailing edge-to-trailing edge; green line (second line from the right): leading edge-to-trailing edge; blue line (rightmost line): trailing edge-to-leading edge.

**Figure 3:**
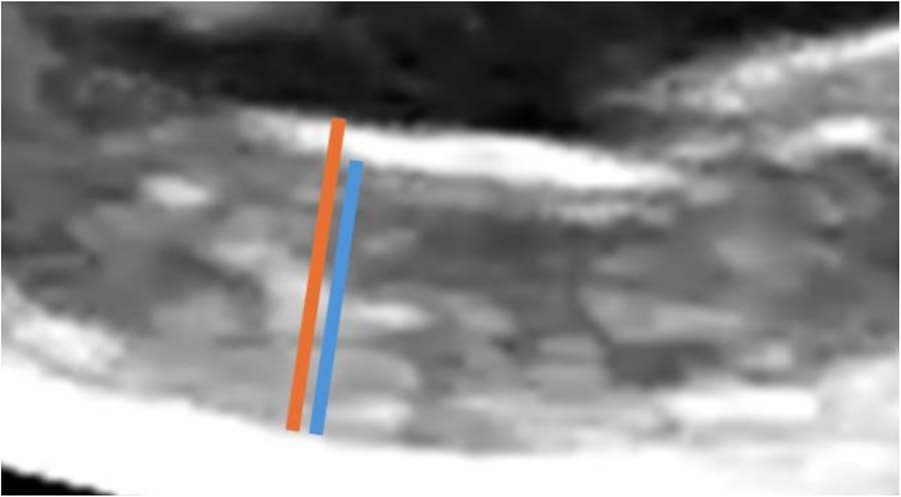
Magnification of the left ventricular free wall showing the different measurement techniques. Orange line on the left: leading edge-to-leading edge; blue line on the right: trailing edge-to-leading edge.

### Statistical analysis

Data were tested for normality graphically and by using a Shapiro–Wilk test. Normally distributed data are reported as mean ± SD and non-normally distributed data as median (interquartile range [IQR]). Categorical data were presented as frequency and percentage. Comparisons among the 3 groups (healthy cats, equivocal cats, and cats diagnosed with HCM) were performed using one-way analysis of variance (ANOVA) for continuous parametric data and the Kruskal–Wallis test for continuous nonparametric data. A χ-squared test was used to compare categorical variables among the 3 groups.

Measurement variability and reliability were analyzed using coefficients of variation, 95% repeatability/reproducibility coefficients and intraclass correlation coefficient (ICC).

Differences between the measurement techniques were tested using Friedman’s test for IVS thickness including pairwise Wilcoxon signed-rank tests with Bonferroni correction and solely Wilcoxon signed-rank for LVFW.

The agreement of different measurement techniques leading to the diagnosis of HCM was tested using Cohen’s κ. Agreement of continuous measurements, including different endocardial borders, was assessed using Bland–Altman analysis.

A detailed description of Materials and Methods is added as an appendix to this paper.

## Results

### Study cohort

A total of 367 cats were presented to the Queen Mother Hospital for Animals Cardiology Service for echocardiograms between January 2023 and February 2024. A diagnosis of HCM was made in 153 (41.6%) cats; 46 (12.5%) cats were diagnosed as equivocal, and 94 (25.6%) cats were diagnosed as normal. The prevalence of HCM in this population is higher than previously reported in referral and non-referral populations but only includes cats referred to the Cardiology Service of a tertiary referral center. Of these cats, 131 met the inclusion criteria to be included in the study. There were 42 (32.06%) cats with normal LVWT, 29 (22.13%) cats with equivocal wall thickness, and 60 (45.8%) cats diagnosed with HCM phenotype. From this group of cats meeting the inclusion criteria, 17 cats with diagnosed normal LVWT, 17 cats with equivocal wall thickness, and 18 cats with an HCM phenotype were randomly chosen (Figure 4). The median age was 7.0 [IQR, 0.9-15.1] years, and mean body weight was 4.5 ± 0.97 kg. Thirty-seven of 52 (71%) cats were male, and 41/52 (79%) cats were non-pedigreed. Of the included pedigreed cats, 3 Persians and 2 British Shorthair cats were included, as well as one of the each of the following breeds: Bengal, Birman, British Longhair, Maine Coon, Siamese, Sphynx. Clinical data are summarized in Table 1.

**Figure 4:**
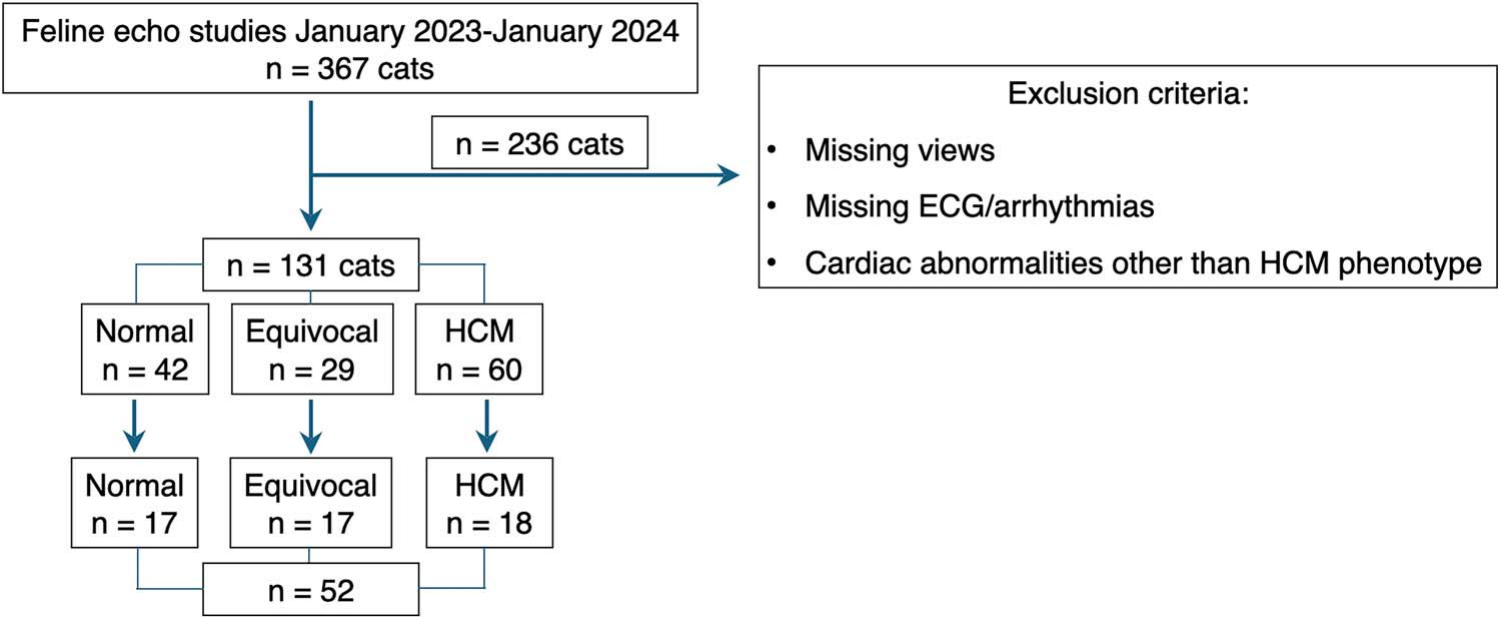
Flow chart representing steps of patient selection.

**Table 1:** Clinical characteristics of the study sample.

Variable	All (*n* = 52)	Normal (*n* = 17)	Equivocal (*n* = 17)	HCM phenotype (*n* = 18)	*P* value
**Age (years)**	7.0 [2.8-11.2]	4.3 [1.9-11.1]	6.5 [2.3-10.7]	8.0 [3.7-12.3]	.1
**Body weight (kg)**	4.5 (±0.97)	4.5 (±1.1)	4.4 (±1.05)	4.5 (±0.76)	.2
**Sex (male/female)**	37/15	13/4	11/6	13/5	.8
**BCS (/9)**	4.5 (2-7)	3.9 (2-5)^a^	4.3 (2-6) ^a,b^	5.1 (4-7)^b^	**.009**
**Non-pedigree**	41 (78%)	12 (70%)	13 (76%)	16 (88%)	.3

Normally distributed data are presented as mean (±SD), non-normally distributed data are presented as median [interquartile range]. Categorical data are presented as frequency and percentage. For BCS, superscript pairs sharing the same superscript letter are not significantly different from each other (^a^*P* = .9, ^b^*P* = .1).

Abbreviations: BCS = body condition score; LVWT = left ventricular wall thickness.

### Echocardiographic data

Echocardiographic data are summarized in Table 2. Measurements for the entire cohort measured by a diplomate in cardiology are shown in Figures 5 and 6. IVS wall thickness was significantly larger for the L-T measurement (5.5 [IQR, 3.8-7.2] mm) compared with other methods (*P* < .001), whereas the T-L method produced significantly smaller results (4.3 [IQR, 2.8-5.8] mm for the IVS and 4.8 [IQR, 3.4-6.2] mm for the LVFW) compared with other methods (*P* < .001) for both IVS and LVFW thickness. When exclusively assessing healthy cats, equivocal cats, or cats with HCM, T-L produced consistently smaller LVWT measurements compared with other methods (healthy cats, *P* < .001 for IVS thickness and LVFW; equivocal cats, *P* < .001 for IVS thickness and LVFW; HCM cats, *P* < .001 for IVS and LVFW).

**Table 2:** Median and [interquartile range] of left ventricular wall thickness at end-diastole measured using different endocardial borders measured by the board-certified cardiologist.

Variable	Leading-edge to leading-edge	Leading-edge to trailing-edge	Trailing-edge to leading-edge	Trailing-edge to trailing-edge
**Whole cohort: 52 cats**				
**IVS (mm)**	4.9 [3.4-6.4]	5.5^a^ [3.8-7.2]	4.3 [2.8-5.8]	4.8^a^ [3.6-6.0]
**LVFW (mm)**	5.2 [3.8-6.6]		4.8 [3.4-6.2]	
**Normal: 17 cats**				
**IVS (mm)**	4.3 [3.6-5.0]	4.8^b^ [4.2-5.5]	3.7 [3.0-4.3]	4.2^b^ [3.5-4.9]
**LVFW (mm)**	4.2 [3.5-5.0]		3.8 [3.1-4.5]	
**Equivocal: 17 cats**				
**IVS (mm)**	4.8 [3.9-5.7]	5.5^c^ [4.5-5.5]	4.3 [3.4-5.2]	4.7^c^ [3.7-5.7]
**LVFW (mm)**	4.9 [3.7-6.1]		4.6 [3.5-5.7]	
**HCM phenotype: 18 cats**				
**IVS (mm)**	5.5 [4.3-6.7	6.2^d^ [5.0-7.4]	4.9 [3.5-6.3]	5.5^d^ [4.2-6.8]
**LVFW (mm)**	6.4 [5.0-7.8]		5.9 [4.4-7.4]	

Within a line, pairs sharing the same superscript letter are not significantly different from each other. ^a^*P* = .3, ^b^  *P* = .2, ^c^  *P* = .6, ^d^  *P* = .9, while all other pairs were significantly different from each other (*P* < .001).

**Figure 5:**
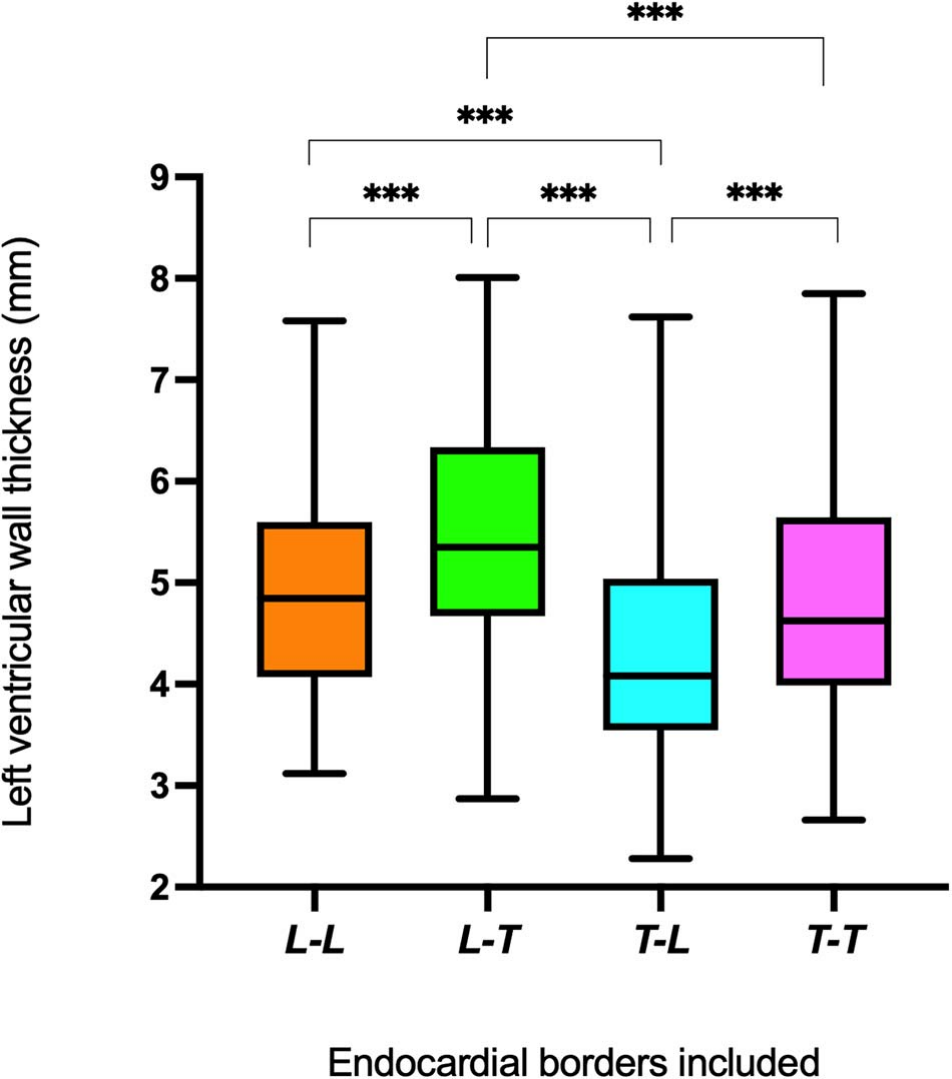
Interventricular septum wall thickness measured in the entire study cohort using different endocardial borders.

**Figure 6:**
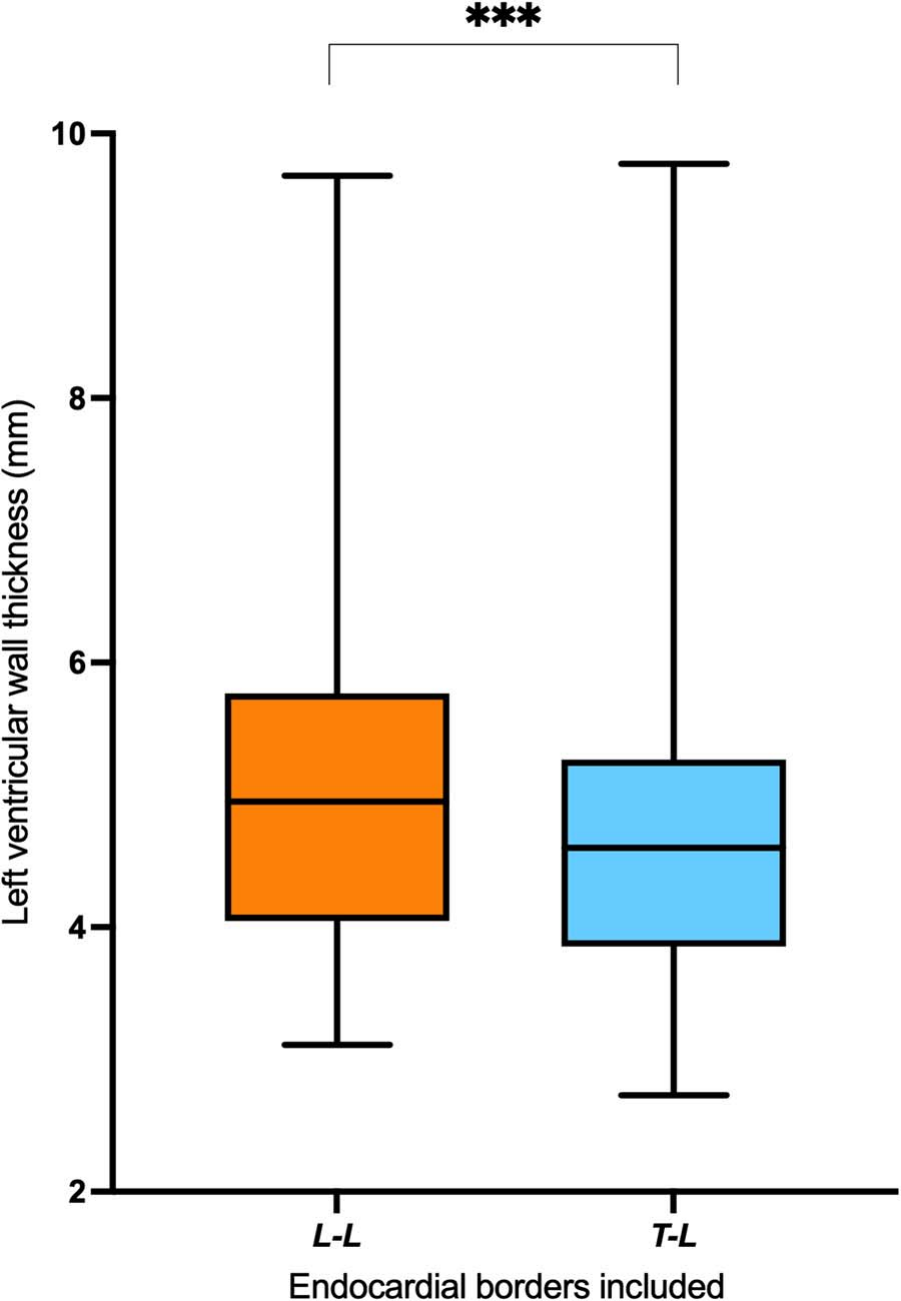
Left ventricular free wall thickness measured on the entire study cohort using different endocardial borders.

Agreement in HCM diagnosis based on measurements of the board-certified cardiologist was considered good for the L-L method compared with the T-L method on the LVFW (κ 0.8). For the IVS, agreement was fair when L-T and T-L methods were compared (κ 0.35), but excellent for L-L and T-T (κ 0.86). Agreement on HCM diagnosis for all other IVS methods was considered moderate to good (L-L and L-T, κ 0.55; L-L and T-L, κ 0.73; L-T and T-T, κ 0.65; T-L and T-T, κ 0.61).

The mixed-model ANOVA based on measurements the cardiology resident and veterinary student performed showed that observer and measurement technique were significant (*P* < .001) sources of variability for IVS thickness and LVFW measurements, but not for different timepoints (day 1 vs day 14). The within-subject correlation across repeated measurements was modeled using an autoregressive (AR 1) covariance structure. The random subject accounted for 16% of the total variance (ICC 0.84).

Agreement of the different methods assessed by Bland Altman analysis showed the narrowest 95% limits of agreement (LoA) for T-L measurement of the IVS (95% LoA, −0.58 to 0.67 mm; mean bias, 0.04 mm; beta1, 0.5; *P* = .22) by the cardiology resident and the narrowest 95% LoA for the L-T method by the veterinary student for the IVS (95% LoA, −1.2 to 1.17 mm; mean bias, −0.01 mm; β1, −0.22; *P* = .13). Overall, 95% LoAs were wider for the veterinary student (LL: 95% LoA, −1.25 to 1.16 mm; mean bias, -0.05 mm; β1, -0.22; *P* = .11; TL: 95%LoA, −1.34 to1.33 mm; mean bias, −0.01 mm; β1, −0.21; *P* = .14; TT: 95% LoA, −1.25 to 1.21 mm; mean bias, −0.02 mm; β1, −0.21; *P* = .15) than for the cardiology resident (LL: 95% LoA, −0.76 to 0.84 mm; mean bias, 0.04 mm; β1, −0.3; *P* = .94; LT: 95% LoA, −0.7 to 0.87 mm; mean bias, 0.08 mm; β1, −0.19; *P* = .63; TT: 95% LoA, −0.68 to 0.7 mm; mean bias, 0.01 mm; β1, 0.08; *P* = .82) for the IVS. For LVFW measurements, mean bias was—0.12 and −0.47 mm with 95% LoA, −1.18 to 0.94 mm; β1, −0.15; *P* = .60 and −1.2 to 0.79 mm; β1, 0.05; *P* = .89 for L-L and T-L, respectively, for the cardiology resident. The veterinary student measurements showed a mean bias of 0.07 and 0.06 mm with 95% LoA, −0.93 to 1.08 mm; β1, −0.6; *P* = .10 and 95% LoA, −0.91 to 1.03 mm; β1, −0.6; *P* = .11 for L-L and T-L, respectively.

Interobserver agreement assessed by Bland-Altman analysis also showed wide LoA. For measurement of the IVS, L-L and T-T methods both had the narrowest 95% LoA (LL, −1.55 to 0.73 mm; TT, −1.74 to 0.53 mm) with a mean bias of −0.41 and −0.62 mm and β1, −0.02, *P* = .76 and β1, −0.05; *P* = .44, respectively. The LT measurement of the IVS showed 95% LoA, −1.43 to 0.92 mm; mean bias, −0.25; β1, 0.003; *P* = .96 and TL measurements had 95% LoA, −1.89 to 0.5 mm; mean bias, −0.7 mm; β1, 0.018; *P* = .81. Measurement of the LVFW showed 95% LoA of −1.63 to 1.05 mm; β1, −0.18; *P* = .09 and 95% LoA of −1.85 to 0.4 mm with mean bias of 0.38 and 0.72 mm; β1, −0.15; *P* = .91 for L-L and T-L method, respectively.

Intraobserver repeatability and interobserver reproducibility measurements are summarized in Tables 3 and 4. Coefficients of variation (CV) for all measurements were <10% for the veterinary student, indicating excellent repeatability. The CV for the veterinary student were overall higher than for the cardiology resident, but all were <10% except for the L-L method for IVS thickness measurement (CV, 10.3%). The interobserver CV was higher than intraoperator CVs, ranging from 8 (L-T method for IVS) to 16.7 (T-L method for LVFW) for the board-certified cardiologist and the cardiology resident.

**Table 3:** Intraobserver variability for inclusion of different endocardial borders on the interventricular septum and left ventricular free wall.

		CV		95% RC		ICC	
		Intra-obs (op 1)	Intra-obs (op 2)	Intra-obs (op 1)	Intra-obs (op 2)	Intra-obs (op 1)	Intra-obs (op 2)
**IVS**	LL	4.5	10.3	0.78	2.09	0.97	0.93
	LT	3.9	4.1	0.78	1.18	0.96	0.94
	TL	3.6	5.2	0.61	1.35	0.97	0.91
	TT	3.8	4.5	0.68	1.23	0.98	0.93
**LVFW**	LL	5.7	4.9	1.06	1.01	0.94	0.8
	TL	4.4	5.4	1.01	0.99	0.97	0.97

Abbreviations: op 1 = resident in cardiology; op 2 = veterinary student; CV = coefficient of variation; 95% RC = 95% repeatability/reproducability coefficient; ICC = Intraclass correlation coefficient; LL = leading edge-to-leading edge; LT = Leading edge-to-trailing edge; TL = trailing edge-to-leading edge; TT = trailing edge-to-trailing edge; IVS = interventricular septum wall thickness; LVFW = left ventricular free wall thickness.

**Table 4:** Interobserver variability between board-certified cardiologist and cardiology resident for inclusion of different endocardial borders on the interventricular septum and left ventricular free wall.

		CV	95% RC	ICC
**IVS**	LL	9.6	1.44	0.88
	LT	8.0	1.13	0.80
	TL	13.8	1.85	0.83
	TT	9.2	1.35	0.88
**LVFW**	LL	12.8	1.8	0.81
	TL	16.7	2.1	0.75

Abbreviations: CV = coefficient of variation; 95% RC = 95% repeatability/reproducability coefficient; ICC = Intraclass correlation coefficient; LL = leading edge-to-leading edge; LT = Leading edge-to-trailing edge; TL = trailing edge-to-leading edge; TT = trailing edge-to-trailing edge; IVS = interventricular septum wall thickness; LVFW = left ventricular free wall thickness.

For IVS thickness, 95% repeatability coefficient (95% RC) was smaller for the cardiology resident (range, 0.61-0.78 mm for cardiology resident vs 1.18-2.09 for veterinary student) and slightly smaller for the veterinary student for the LVFW (0.99-1.01 mm for veterinary student vs 1.01-1.06 for cardiology resident). The 95% RC for interoperator variability was higher than intraoperator variability.

Intraobserver repeatability for the cardiology resident was considered excellent (ICC range, 0.94-0.98) and higher compared with the veterinary student for most measurements and equal for T-L of the LVFW (ICC, 0.97). Interobserver ICC was good (range, 0.75-0.88) between the board-certified cardiologist and the cardiology resident.

## Discussion

In our study, we evaluated the effect of including endocardial borders in LVWT measurements. The mean difference between L-T and T-L was 1.2 mm for the IVS and 0.4 mm for the LVFW, respectively, for the board-certified cardiologist. Pairwise comparison of the different measuring techniques showed that all were significantly different except for measurement techniques that only included one endocardial border. This finding confirms our hypothesis that including different endocardial borders has an impact on the reported phenotype of individual cats, and methods are not interchangeable. The mixed model analysis identified operator and inclusion of different endocardial borders as significant for the outcome of being diagnosed with HCM. Cohen’s κ demonstrated only fair agreement on HCM diagnosis for T-L and L-T, emphasizing the clinical impact of the discrepancy in these techniques.

These findings are in agreement with previous studies that have described the clinical consequences of lack of consistency among cardiologists,^4^ but studies reporting the values of LVWT according to which endocardial borders are included have been lacking.

In humans, echocardiography is considered the first-choice diagnostic tool for patients with HCM, but ultrasound-enhancing agents are commonly used because this method increases reproducibility and more closely correlates with magnetic resonance imaging (MRI) assessment.^13^^,^^14^ Cardiac MRI has high spatial resolution and is superior for tissue characterization, enabling precise measurements.^13^

When both methods are compared, echocardiography has been shown to overestimate LVWT compared with MRI measurements.^15^ Cardiac MRI is feasible for LVWT measurement in cats with HCM, with excellent inter- and intraobserver repeatability demonstrated in a previous study.^16^^,^^17^ However, cardiac MRI requires general anesthesia and is more costly and time-intensive compared with echocardiography, making it less feasible for cats.^4^^,^^16^^,^^17^

We chose to focus on 2D echocardiographic measurements in our study. Previous studies have indicated that 2D and M-mode are not interchangeable when assessing LVWT.^9^

Accurate assessment of LVWT requires the fulfillment of several methodological prerequisites. Different echocardiographic views, such as a right parasternal long axis four-chamber view, a right parasternal long axis five-chamber view, and a right parasternal short axis view at the level of the mid-papillary muscle level, all should be utilized for assessment of LVWT because they highlight different regions of the myocardium that could be affected by focal thickening.^1^^,^^4^

The quality of the acquired echocardiographic images is an important factor because it should allow the recognition of focal increases in wall thickness as well as enable the operator to distinguish the left ventricular walls from surrounding structures, such as the tricuspid valve, papillary muscles, moderator band, or false tendons.^18^ In humans, left ventricular short axis views decrease the risk of oblique sectioning of the left ventricular wall and accidentally including any of these structures,^19^ and it was therefore decided to choose this view as a standard view in our study. However, it is recommended that measurements from all three 2D views be included in clinical decision-making.^4^

End-diastole is commonly subjectively assessed^4^ because in clinical surroundings, some cats are easily stressed by the application of an ECG or alcohol applied to improve ECG electrode-skin contact, and a consecutive ECG is therefore not always possible. To standardize our results, we only included patients with a concurrent ECG tracing, which also helped to exclude cats with arrhythmias.

Our study also investigated intraoperator reliability and variability between a resident in veterinary cardiology and a final-year veterinary student with only 2 weeks of training before participating in the study as well as interoperator reliability and variability between a board-certified cardiologist and a cardiology resident. Although interobserver repeatability was good, ICCs were lower and CV and 95% RC were higher compared with intraobserver measurements. The CVs in our study were slightly higher than previous reports of 2D LVWT measurements, which ranged from 3% to 19%^9^^,^^11^^,^^20^ Agreement was assessed using the Bland–Altman method, which showed wide LoA when comparing results of the cardiology resident and veterinary student.

A possible cause for the higher interobserver variation compared with intraobserver repeatability might be that operators were provided with an echocardiography loop of at least 3 consecutive cardiac cycles, but each operator was free to choose a cardiac cycle to measure based on their subjective assessment. Timing in the cardiac cycle was standardized using the peak of the R wave of the concurrent ECG trace.

Intraobserver variability showed excellent CV for all methods, but 95% RC ranged between 0.7 and 1 mm for the cardiology resident, predicting repeated measurements to be within a range of 0.7 to 1 mm with a confidence interval of 95%. This range is considered wide based on the clinical effect a 1 mm difference in LVWT and measurement can have and aligns with findings of previous studies,^9^ emphasizing the need for multiple repeated measurements on every echocardiographic assessment.

The cardiology resident obtained the best intraobserver measurements in our study. This outcome was expected and aligns with previous studies that have assessed intraobserver variability. Despite the relative lack of experience of the veterinary student, the ICCs were still considered excellent, suggesting that measuring 2D images in a right parasternal short axis view is repeatable even for an minimally trained operator.

Our study had some limitations. The veterinary student had only 2 weeks of training in measuring LVWT before participating in the study. This level of inexperience is likely to have influenced the agreement between both observers.

Another limitation is the timing within the cardiac cycle. End-diastole was defined as the peak of the R wave on the concurrently obtained ECG. Although this approach may have led to increased intra-observer and interobserver repeatability, this timing may not represent end-diastole in every case. If there is no concurrent ECG, end-diastole is commonly defined as the frame with the largest left ventricular volume, or the first frame where the mitral valve closes, but this factor was not tested in our study. Our study did not aim to evaluate differences in measurements that were caused by different timings in the cardiac cycle caused by measuring based on ECG versus measuring at the subjective end of diastole; however, when patients are presented for a clinical diagnosis, this factor will be important to consider and could be a subject for further research.

In conclusion, we showed that LVWT measurements in cats increased progressively as 1 or more endocardial borders were included. The magnitude of this difference was sufficiently large to potentially change a diagnosis from healthy to a HCM phenotype. It is important that a consensus be reached in order to standardize the number of endocardial borders included in LVWT measurements in cats.

## Off-label antimicrobial declaration

Authors declare no off-label use of antimicrobials.

## Institutional animal care and use committee or other approval declaration

Authors declare no institutional animal care and use committee or other approval was needed.

## Appendix

### Materials and methods

#### Animals

The database of Royal Veterinary College was retrospectively searched for echocardiograms of cats that were presented to the Cardiology Service for investigation of clinical signs, investigation of an auscultation abnormality, or as part of a health check for cats enrolled in a blood donor program.

Echocardiograms were performed by a diplomate in veterinary cardiology or a cardiology resident at the Queen Mother Hospital for Small Animals. Cats were included if the required view was available and of adequate quality and the video loop included at least 3 cardiac cycles, with a concurrent ECG that showed sinus rhythm. Cats were excluded if they had any congenital or concurrent cardiac disease besides an HCM phenotype or if the quality of the echocardiogram was insufficient, showed artifact or did not allow clear identification of left ventricular walls and endocardial borders. Diagnosis at presentation was based on measurements using a L-L technique for IVS and LVFW. Seventeen cats from the normal wall thickness group and the equivocal wall thickness group and 18 cats from the HCM phenotype group were chosen by a stratified randomization technique using an open-access online source (Urbaniak, G. C., & Plous, S. (2013). Research Randomizer (Version 4.0)) for inclusion into the study.

#### Echocardiographic measurements

Wall thickness of all was measured using a dedicated workstation and proprietary software (Echopac version 113, GE healthcare, Chicago, Illinois, USA). One operator was a veterinary student who was trained to measure LVWT 2 weeks before starting the project by practicing measurements on clinical cases not included in the study. Training was guided by the cardiology resident and included an initial demonstration of measurements, followed by the veterinary student practicing measurements for several hours per day. The second operator was a cardiology resident in the beginning of the second year of residency. The third operator was a board-certified cardiologist. For every randomly selected cat, end-diastolic IVS thickness and LVFW thickness were measured on two-dimensional images of a right parasternal short axis view at the level of the papillary muscles. End of diastole was defined as peak of the R wave of the concurrent ECG. All operators performed their measurements on the same image set but were blinded to the patient’s initial diagnosis and to each other’s results. If more than one right parasternal short axis view at the level of the papillary muscles was available, the best image was chosen subjectively by the resident in cardiology based on appropriate image quality, appropriate view and absence of artifact.

#### Statistical analysis

Differences between the measurement techniques including different endocardial borders were tested using Friedman’s test for IVS thickness including pairwise Wilcoxon signed-rank tests with Bonferroni correction. For LVFW thickness, Wilcoxon signed-rank test was used, as only two different measurement techniques were compared. Friedman’s test and Wilcoxon signed- rank tests were performed for the whole study sample, as well as for individual groups of cats with normal LVWT, cats within equivocal LVWT range and HCM-phenotype cats.

For Cohen’s κ, strength of agreement was defined as: 0.21-0.4 fair; .41-0.60, moderate; .61-0.80 good; 0.81-1.0 excellent as previously reported.^21^

Linear regression was performed to determine if fixed or proportional bias existed. Data of Bland–Altman analysis is presented as mean bias and 95% LoA ± SD.

For 95% repeatability/reproducibility coefficients, the within-subject SD was calculated as the square root of the within-subject variance assessed by 1-way ANOVA. Based on the within-subject, 95%RC was calculated (1.96 × √2 × wSD).

ICC was calculated based on a two-way mixed effects model for absolute agreement on multiple based on mean measurements (*k* = 3). The coefficient of variation (CV) was used to assess variability and was calculated as (standard deviation of the measurements/average of the measurements) ×100.

An ICC > 0.75 and %CV < 10% were considered to indicate excellent measurement agreement and low variability, respectively. A mixed-model ANOVA was performed to assess sources of variation for IVS thickness and LVFW thickness measurements, including observer, measurement technique (inclusion of different endocardial borders) and different time points (day 1 and day 14) as fixed effects and nested within cat as random effect. *P* values <.05 were considered statistically significant.

Statistical analysis was performed by commercially available software (SPSS 30, 2024-2025; GraphPad Prism 10.0, 2025).

## Abbreviations

2D: two-dimensional
95%RC: 95% repeatability/reproducibility coefficient
ANOVA: analysis of variance
CV: coefficient of variation
ECG: electrocardiogram
HCM: hypertrophic cardiomyopathy
ICC: intraclass correlation coefficient
IQR: interquartile range
IVS: interventricular septum
L-L: leading edge-to-leading edge
L-T: leading edge-to-trailing edge
LVFW: left ventricular free wall
LVWT: left ventricular wall thickness
LoA: limits of agreement
mm: millimeters
SD: standard deviation
T-L: trailing edge-to-leading edge
T–T: trailing edge-to-trailing edge
